# Increased seizure sensitivity in pregnant mice with genetic knockdown of acid sensing ion channel 2a is associated with impaired hippocampal inflammatory response

**DOI:** 10.3389/fphys.2022.983506

**Published:** 2022-09-14

**Authors:** Maria Jones-Muhammad, Qingmei Shao, Junie P. Warrington

**Affiliations:** ^1^ Program in Neuroscience, University of Mississippi Medical Center, Jackson, MS, United States; ^2^ Department of Neurology, University of Mississippi Medical Center, Jackson, MS, United States

**Keywords:** ASIC, pregnancy, synaptosomes, eclampsia, microglia, cytokines, GABA, glutamate

## Abstract

Acid sensing ion channels (ASICs) are mechano- and chemo-receptor channels that are activated by drops in extracellular pH as occurs after neurotransmission. In our previous study, we demonstrated that mice subjected to reduced utero-placental perfusion pressure during pregnancy, to mimic the pregnancy complication of preeclampsia, have reduced hippocampal expression of ASIC2a protein. We also showed that pregnant mice with heterozygous expression of ASIC2a (+/-) had increased sensitivity and severity to pentylenetetrazol-induced seizures; however, the mechanisms by which this occurs remain unclear. The purpose of this study was to investigate key molecular targets involving neurotransmission and inflammation that are differentially changed following seizure exposure in pregnant ASIC2a +/- mice. On gestational day 18.5, ASIC2a wild-type (+/+, *n* = 7) and +/- (*n* = 14) mice were injected with 40 mg/kg pentylenetetrazol and monitored for 30 min. Western blot and ELISA analysis revealed no difference in hippocampal synaptosome glutamate-related proteins but an increase in GABA concentration in pregnant +/- mice. Using ELISA and multiplex assays, we found a significant decrease in serum TNFα, and a decreased concentration of pro-inflammatory cytokines and chemokines in hippocampal cytosolic fraction. Significant reductions in IL-1β, IL-3, IL-12 (p70), eotaxin, interferon gamma, and macrophage inflammatory protein (MIP-1β), in the hippocampal cytosolic fractions of +/- mice were observed compared to +/+ mice. Additionally, there was no difference in hippocampal microglia density or activation in pregnant ASIC2a+/+ vs. +/- mice. These results support the hypothesis that pregnant mice with reduced ASIC2a may not be able to mount an inflammatory response following acute seizure exposure.

## Introduction

Eclampsia, the occurrence of first-time seizures during pregnancy in women with no history of seizure disorders, can be deadly to both the mother and the developing fetus (Das and Biswas, 2015). Additionally, epilepsy patients have increased risk of mortality and adverse complications during pregnancy (MacDonald et al., 2015). To date, the mechanisms that contribute to novel seizures during pregnancy are still unclear. In our previous study, we showed that the preclinical mouse model of reduced utero-placental perfusion (RUPP), which shares some of the clinical features of preeclampsia, has decreased placental and hippocampal expression of acid sensing ion channel 2a (ASIC2a) (Jones-Muhammad et al., 2021). ASICs are mechano- and chemo-receptor channels with important roles in modulating seizure activity (Liang et al., 2015). Both ASIC1a and ASIC3 have been shown to be important for seizure termination (Ziemann et al., 2008; Cao et al., 2016; Cao et al., 2018). Interestingly, in our previous publication, we showed that pregnant ASIC2a heterozygous knockout (+/-) mice have increased seizure severity and sensitivity (Jones-Muhammad et al., 2021). The molecular mechanisms underlying increased seizure sensitivity and severity in pregnant ASIC2a +/- mice are not fully known. Additionally, the acute effects of seizure exposure on pregnant mice with wild-type or heterozygous expression of ASIC2a have not been fully detailed. Two pathways that are involved in seizure pathophysiology are dysregulated neurotransmitter activity and neuroinflammation.

It is widely accepted that dysregulation of excitatory and inhibitory neurotransmitter expression and activity is associated with increased likelihood of seizures (Huguenard, 2003). Indeed, several studies have indicated that increased excitation due to increased glutamate with either no change or a reduction in GABA concentration, contributes to increased seizure activity (During and Spencer, 1993; Rowley et al., 1997; Cavus et al., 2016; Dhaher et al., 2021). Moreover, previous work also indicates that neurotransmitter transporters are involved in seizure activity, where Mathern and colleagues reported that temporal lobe epilepsy patients have abnormal changes in the expression of both presynaptic and astrocytic transporters for GABA and glutamate (Mathern et al., 1999). Because ASICs participate in neurotransmission (Shteinikov et al., 2022), it is possible that neurotransmitter activity is significantly affected by reductions in ASIC2a and following seizure activity.

Systemic and neuro-inflammation increase the risk of seizures (Vezzani et al., 2011) and are affected by seizure activity. Using an animal model of epileptogenesis, Simoni and colleagues showed increased neuroinflammation, in the form of increased markers such as IL-1 beta and tumor necrosis factor (TNF)-alpha (De Simoni et al., 2000). Interestingly, ASICs have been suggested to play a critical role in neuroinflammation. Previous work by Yu and colleagues demonstrated that inflammation induced by lipopolysaccharide resulted in increased expression of both ASIC1 and ASIC2a in rat microglia (Yu et al., 2015), suggesting a role in microglia activation. The study did not test the specific role ASIC2a may have in seizure-induced inflammation. It is even less clear whether increased inflammation may be a contributing factor to worse seizures in pregnant mice with reduced expression of ASIC2a.

In this study, we measured changes in hippocampal glutamate and GABA concentration and expression of some of the related receptors and transporters in hippocampal synaptosomes from pregnant ASIC2a mice following acute seizure activity. We also assessed changes in inflammatory cytokines and chemokines in serum and hippocampus and quantified hippocampal microglia density and activation following seizure activity.

## Materials and methods

### Animals

Heterozygous B6.129-Asic2tm1Wsh/J mice were obtained from Jackson Laboratory (JAX Mice stock number: 013126, RRID: IMSR_JAX:013126) (Wu et al., 2012) and bred in the Lab Animal Facilities at the University of Mississippi Medical Center (UMMC) to generate genotypes for the experiments. Mice were fed a standard rodent diet (Teklad 22/5 Rodent Diet 8640) and water *ad libitum* until breeding, when they were switched to a breeders’ diet (Teklad global 19% protein extruded rodent diet 2019) from mating until euthanasia at Gestational day (GD) 18.5. Mice were housed two to five per cage at a constant temperature of ∼74 ℉ and a 12 h light and 12 h dark cycle. All procedures were approved by the UMMC Institutional Animal Care and Use Committee (Protocol: 1434 and 1434A) in accordance with the 8th edition of the National Research Council Guide for Care and Use of Laboratory animals.

### Acid sensing ion channel 2a genotyping

ASIC2a genotyping method is described elsewhere (Jones-Muhammad et al., 2021). Genotyping was performed using ear punches at weaning and tail snips at the end of the experimental protocol to confirm ASIC2a genotype. The KAPA Mouse Genotyping Kit from KAPABIOSYSTEMS was used for DNA extraction and PCR genotyping. The protocol for using KAPA Expression Extract required a 50 µl solution comprised of 44 µl of di-deionized water, 5 µl of 10X KAPA Expression Extract Buffer, and 1 U/µl of KAPA E. E. Enzyme. The tail snip was added to the solution, and the tissue was lysed at 75°C for 10 min. Enzyme was inactivated at 95°C for 5 min. After lysis, the lysate was centrifuged for 5 min and the supernatant was stored for genotyping. The following sequences were used (5′-3′): Reaction A: AGT CCT GCA CGG TGG GAG CTT CTA; GAA GAG GAA GGG AGC CAT GAT GAG, Reaction B: ATG GTT TCG GAG TGG TTT GGC ATT GTG; TGG ATG TGG AAT GTG TGC GA. Samples were run on 2% gels comprised of 35 ml of di deionized water, 0.7 g of agarose gel, and 3.5 µl of GelRed. 6 µl of 100 bp ruler was loaded and 5 µl of sample was loaded before running the gel at 140 V for 45 min. Gels were imaged using the ChemiDoc MP system. The heterozygous mice produce bands that migrates to 365 and 450 bp, and the wild-type band is 365 bp.

### Breeding and establishment of timed pregnant acid sensing ion channel 2a mice

One to three female mice were paired with one male of the same age and genotype in the evening. The following day, male mice were returned to their home cages. As mice were paired for only one night, the exact date of pregnancy is known. The day of separation was considered as GD 0.5. Mice were monitored for changes in abdominal size, and pregnancy was confirmed on GD 13.5. Mice that did not become pregnant were subjected to one additional round of mating.

### Seizure induction

Seizures were induced using methods mentioned elsewhere (Jones-Muhammad et al., 2021). On GD 18.5, pregnant ASIC2a wild-type (+/+) (*n* = 7) and heterozygous (+/-) (*n* = 14) mice were administered 40 mg/kg of pentylenetetrazol (PTZ) *via* intraperitoneal (i.p.) injection. After injection, mice were video monitored for 30 min. The observer was blind to the genotype. Behavior was scored using a revised version of the Racine seizure scoring scale (Kolosowska et al., 2016) from 0 to 7, with 0 indicating no seizure or normal behavior and seven indicating respiratory arrest and death. Seizure activity was analyzed using the Observer XT software (Noldus Information Technology, Leesburg, VA). We measured latency to the first seizure behavior, the total duration of seizure activity, the total duration of and latency to severe seizures (score 4–7), and the highest seizure score of each animal throughout the behavior monitoring. Immediately after observation, mice were euthanized and organs harvested under isoflurane anesthesia.

### Euthanasia and tissue processing

At gestational day (GD) 18.5, after seizure monitoring, mice were weighed and anesthetized using 3% isoflurane (SomnoSuite, Kent Scientific), and blood was collected *via* cardiac puncture. Organs (heart and kidneys) were excised and weighed. The number of live and resorbed pups were counted, and pups and placentas were weighed. The mean pup and placenta weight were calculated per dam. Blood samples were used for assessing hematocrit, which is a crude indicator of systemic hypoxia (Wu et al., 2016). Brains were removed and hemisected (after removal of cerebellum). The right hemisphere was transferred to 4% paraformaldehyde, followed by 30% sucrose for 72 h. Right hemisphere was embedded in cryogel and frozen until further processing. The left hemisphere was dissected to isolate the hippocampus, which was flash frozen in liquid nitrogen and stored at −80°C until further processing.

### Isolation of synaptosomal and cytosolic fractions from hippocampus

The method for Synaptosome isolation was adapted from a previous protocol (Wu et al., 2012) Samples were homogenized using Syn-PER™ Synaptic Protein Extraction Reagent (ThermoFisher, #87793) in a mini bead homogenizer at 4,000 rpm. Homogenates were centrifuged at 1,200×g for 10 min at 4°C. After centrifugation, the supernatant was placed in a second tube and centrifuged at 15,000×g for 20 min at 4°C. The supernatant was collected as the cytosolic fraction and the pellet containing synaptosomes was resuspended in 1X phosphate buffered saline (PBS) and stored at −80°C until later processing. Bicinchoninic acid (BCA) protein assay was used to determine the amount of protein in each sample following manufacturer’s directions.

### ELISA and neurotransmitter assay analysis

Using commercially available ELISA kits, we measured serum TNFα (R&D Systems, MTA00B) and IL-17 (R&D Systems, M1700) in duplicates following manufacturer’s directions. To assess glutamate levels, synaptosome samples were run on the glutamate fluorometric assay kit (abcam, ab138883). Synaptosome GABA was measured using the general GABA ELISA kit (MyBioSource, MBS2700393). Samples were run in duplicates and normalized to protein concentration.

### Western blot analysis

Hippocampal synaptosomal samples were prepared for Western blot analysis using 1X PBS and 4X sample buffer containing 2 beta-mercaptoethanol. Samples were electrophoresed using Criterion TGX 4-20% Stain-free gel at 120 V for 90 min. Once complete, the gel was activated using ChemiDoc MP Imaging System for 45 s. Trans blot turbo transfer kit was used to transfer protein gels onto nitrocellulose membranes. After 30 min of blocking in Odyssey blocking buffer, primary antibodies of interest were added and incubated at 4°C overnight. A list of each primary antibody, along with other relevant information is found on Table 1. After washing, secondary antibodies were added and membranes were imaged using ChemiDoc MP system. Secondary antibodies used are also found on Table 1. Target protein expression was normalized to alpha tubulin expression and blots were analysed using Image Lab software (Bio-Rad).

**TABLE 1:** List of primary and secondary antibodies used for Western blot analysis.

Antibody target	Host	Dilution	Company	Catalogue #	RRID
NMDAR1	Rabbit	1:1,000	abcam	ab17345	AB_776808
NMDAR2b	Rabbit	1:1,000	abcam	ab65783	AB_1658870
VGLUT	Rabbit	1:1,000	abcam	ab227805	AB_2868428
EAAT1	Rabbit	1:1,000	abcam	ab416	AB_304334
EAAT2	Rabbit	1:1,000	abcam	ab41621	AB_941782
GABA_A_R	Rabbit	1:1,000	abcam	ab33299	AB_732498
GAT1	Rabbit	1:1,000	abcam	ab426	AB_2189971
α-Tubulin	Chicken	1:15,000	abcam	ab89984	AB_10672056
Anti-Rabbit (800,680)	Donkey	1:15,000	Li-Cor	925-32213	AB_2715510
				925-68073	AB_2716687
Anti-Chicken (800,680)	Donkey	1:15,000	Li-Cor	925-32218	AB_2814922
				925-68028	AB_2814923

### Multiplex cytokine/chemokine measurement

Bio-Plex Pro Mouse Cytokine 23-plex assay (Bio-Rad, M60009RDPD) was used to compare the concentration of inflammatory cytokines and chemokines in hippocampal cytosolic fractions from ASIC2a +/+ and +/- pregnant mice. Samples were thawed and diluted 1:2 with Bio-Plex sample diluent and run following manufacturer’s directions. Standard curves were generated for each analyte and sample concentrations determined. All measured concentrations were normalized to protein concentration in each sample.

### Immunofluorescence analysis of hippocampal microglia

.The right hemisphere was fixed in 4% paraformaldehyde for 24 h and transferred to 30% sucrose. Hemispheres were embedded in Cryo-Gel and frozen on dry ice. Embedded brains were stored at −80°C until processing. Brains were cryo-sectioned at 20 μm thickness and transferred to frosted slides for immunofluorescence staining. Slices were blocked using 10% normal donkey serum and 5% Bovine Serum Albumin, followed by overnight incubation in primary antibodies: 1:1,500 rabbit anti-Iba1 (Ionized calcium binding adaptor molecule one; Wako 019-19741) and 1:1,000 mouse anti-MHCII (major histocompatibility complex II; abcam ab23990). Secondary antibodies were 1:600 Rhodamine-conjugated donkey anti-rabbit Jackson ImmunoResearch Lab; 711-025-152 and 1:1,000 Alexa Fluor 488 donkey anti-mouse (Jackson ImmunoResearch Lab; 715-545-150). Z-stacks (512 × 512 pixels) were captured using confocal microscopy (Nikon) at 0.5 μm steps using a 60X oil objective lens in the CA1, CA3, and DG of each brain section. The number of microglia in each region was counted by an investigator blinded to the genotype. Microglia density (per mm^2^) was calculated and averaged per hippocampus per mouse. Maximum projection images of the z-stacks are shown for the representative images.

### Statistical analysis

Normality of data was assessed using the Shapiro-Wilk test. We used Mann Whitney *U* tests for non-normal data distributions and unpaired *t* test for normally distributed data. Data are presented as mean ± SD. Differences were considered statistically significant at the *p* < 0.05 level. Statistical analyses were performed and graphs were generated using GraphPad Prism software (version 9.0.0).

## Results

### General characteristics and pregnancy outcomes

**FIGURE 1:**
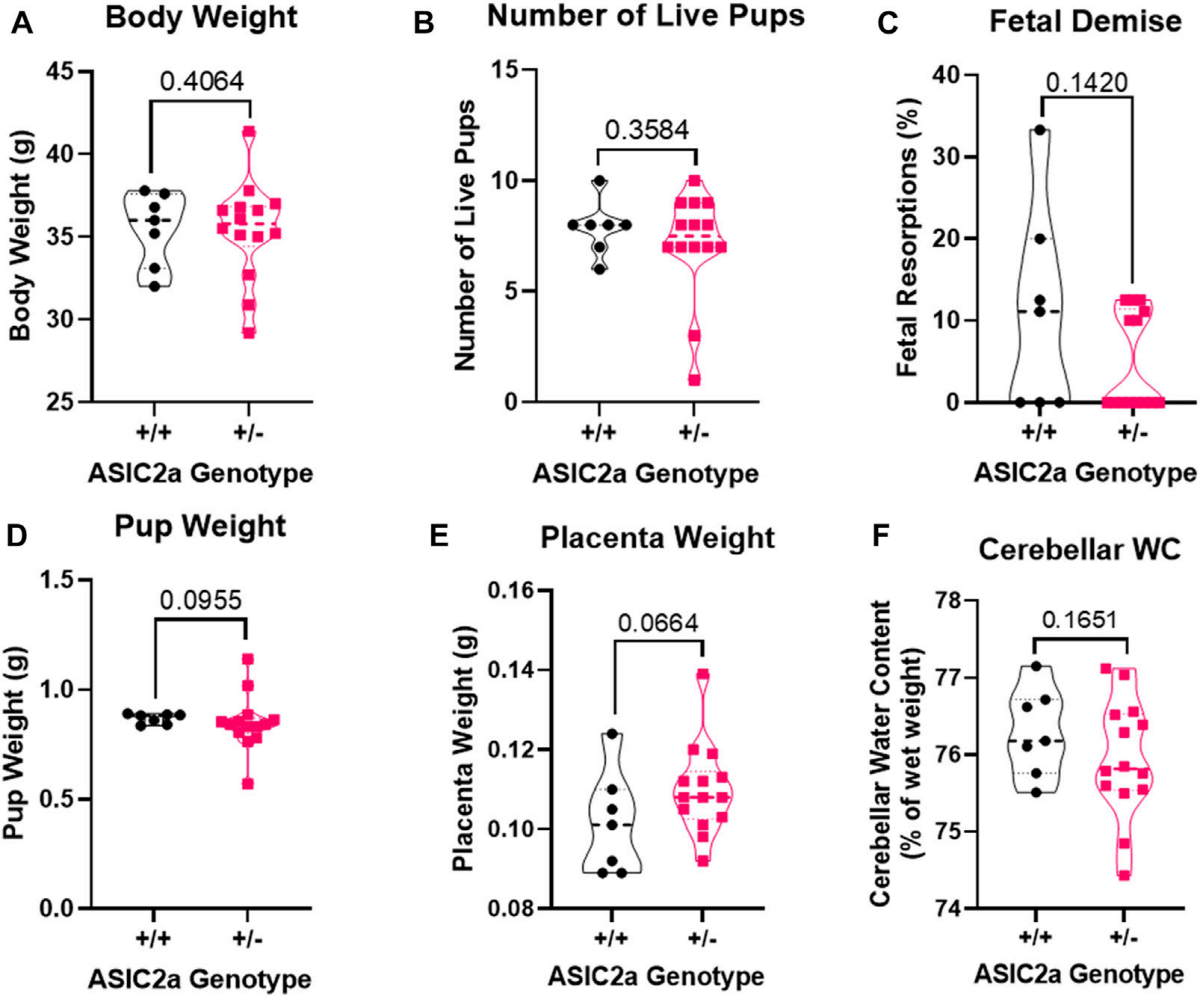
General pregnancy characteristics of mice at gestational day 18.5 of pregnancy. There was no difference in **(A)** body mass, **(B)** number of live pups *in utero*, **(C)** fetal demise, **(D)** average pup weight, **(E)** average placental weight, or **(F)** cerebellum water content between ASIC2a +/+ and +/- pregnant mice. N = 7 or 14 per genotype. Individual data points are displayed. Thick dashed line is the median and the thin dashed lines are the quartiles. Significance was calculated using Mann-Whitney *U* test.

As reported in our previous publication (Jones-Muhammad et al., 2021) and shown in Figure 1, at gestational day 18.5 of pregnancy, there was no difference in maternal body weight (35.5 ± 2.2 g in +/+ vs. 35.4 ± 3.0 g in +/-, Figure 1A), number of live fetuses *in utero* (8 ± 1 in +/+ vs. 7 ± 2 in +/-, Figure 1B), percent resorbed fetuses (11.0 ± 12.5% in +/+ vs. 4.9 ± 5.9% in +/-, Figure 1C), mean fetal weight (0.87 ± 0.02 g in +/+ vs. 0.85 ± 0.13 g in +/-, Figure 1D), placenta weight (0.10 ± 0.01 g in +/+ vs. 0.11 ± 0.01 g in +/-, Figure 1E), and cerebellar water content (76.29 ± 0.57% vs. 75.95 ± 0.77% in +/-, Figure 1F).

### Reduced acid sensing ion channel 2a and increased seizure severity is not associated with changes in excitatory neurotransmitter-related proteins at the synapse

**FIGURE 2:**
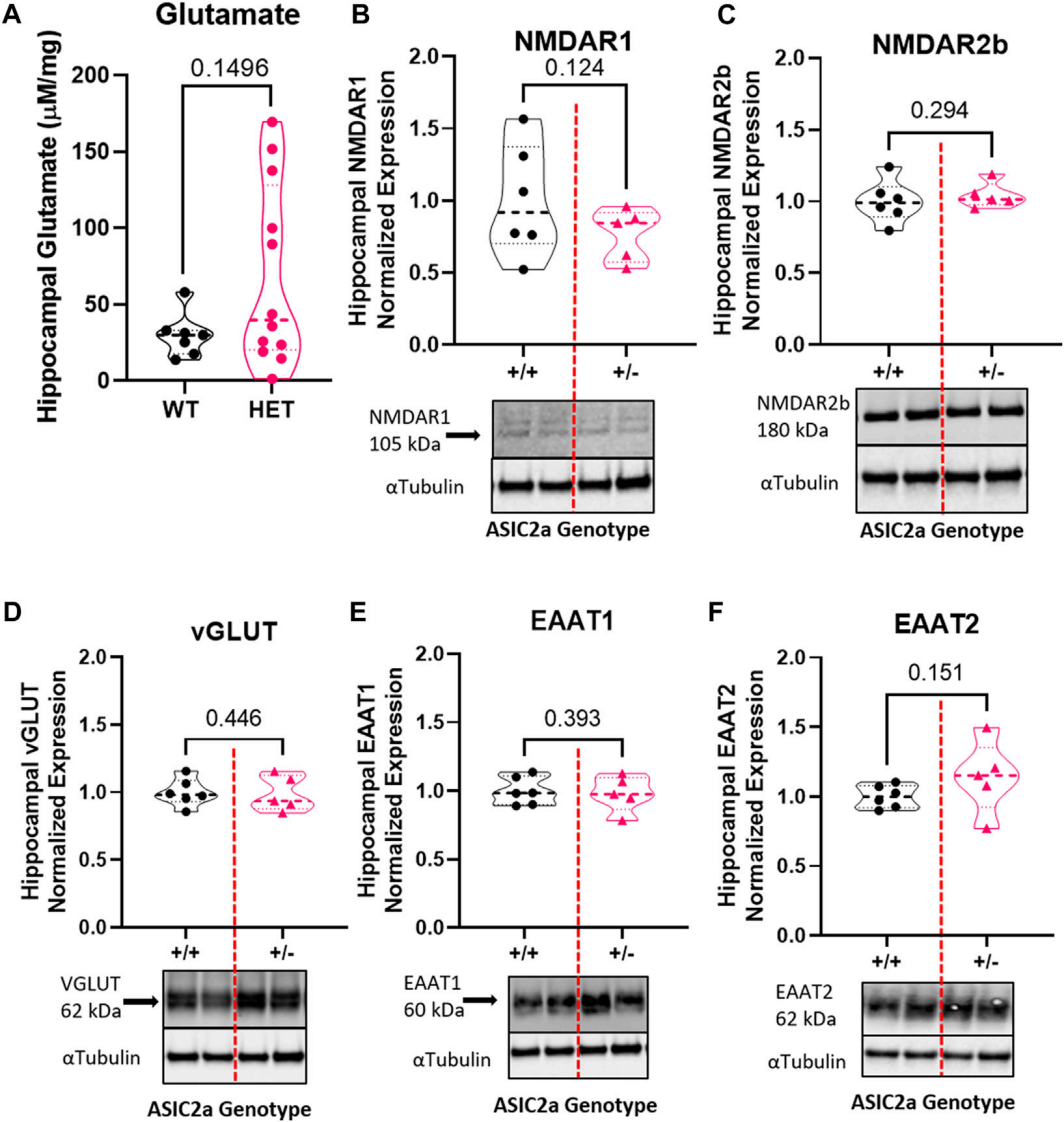
Changes in excitatory neurotransmitter-related proteins in hippocampal synaptosomes following seizures in pregnant mice with reduced ASIC2a. No difference in synaptosomal levels of **(A)** glutamate, **(B)** NMDAR1, **(C)** NMDAR2b, **(D)** EAAT1, **(E)** EAAT2, or **(F)** vGLUT following seizures in pregnant ASIC2a heterozygous mice. NMDAR - N-methyl-D-aspartate receptor, EAAT—excitatory amino acid transporter, vGLUT—vesicular glutamate transporter. N = five to six mice per genotype. Individual data points are displayed. Thick dashed line is the median and the thin dashed lines are the quartiles Significance was calculated using Unpaired *t*-test.

To determine whether seizure exposure in pregnant ASIC2a +/- mice resulted in increased excitatory neurotransmitter activity at the synapse, we isolated synaptosomes from hippocampal samples and measured glutamate concentration. There was no difference in hippocampal synaptosome glutamate concentration (29.8 ± 14.4 vs. 130.1 ± 232.2 μM in +/- mice; Figure 2A). Next, we assessed protein expression of NMDA receptors and found no difference in NMDAR1 (1.0 ± 0.39 in +/+ vs. 0.77 + 0.18 in +/-; Figure 2B) or NMDAR2b (1.0 ± 0.15 in +/+ vs. 1.04 ± 0.09 in +/-; Figure 2C) in hippocampal synaptosomes. There was no difference in vesicular glutamate transporter expression (VGLUT) (1.0 ± 0.10 in +/+ vs. 0.99 ± 0.13 in +/-; Figure 2D). Once released, glutamate is rapidly taken up by astrocytes and neurons *via* transporters; therefore, we assessed changes in EAAT expression. There was no significant difference in EAAT1 (1.0 ± 0.10 in +/+ vs. 0.98 ± 0.13 in +/-; Figure 2E) or EAAT2 (1.0 ± 0.08 in +/+ vs. 1.14 ± 0.26 in +/-; Figure 2F) expression in hippocampal synaptosomes.

### Seizure activity increases hippocampal GABA concentration in pregnant mice with reduced ASIC2a

**FIGURE 3:**
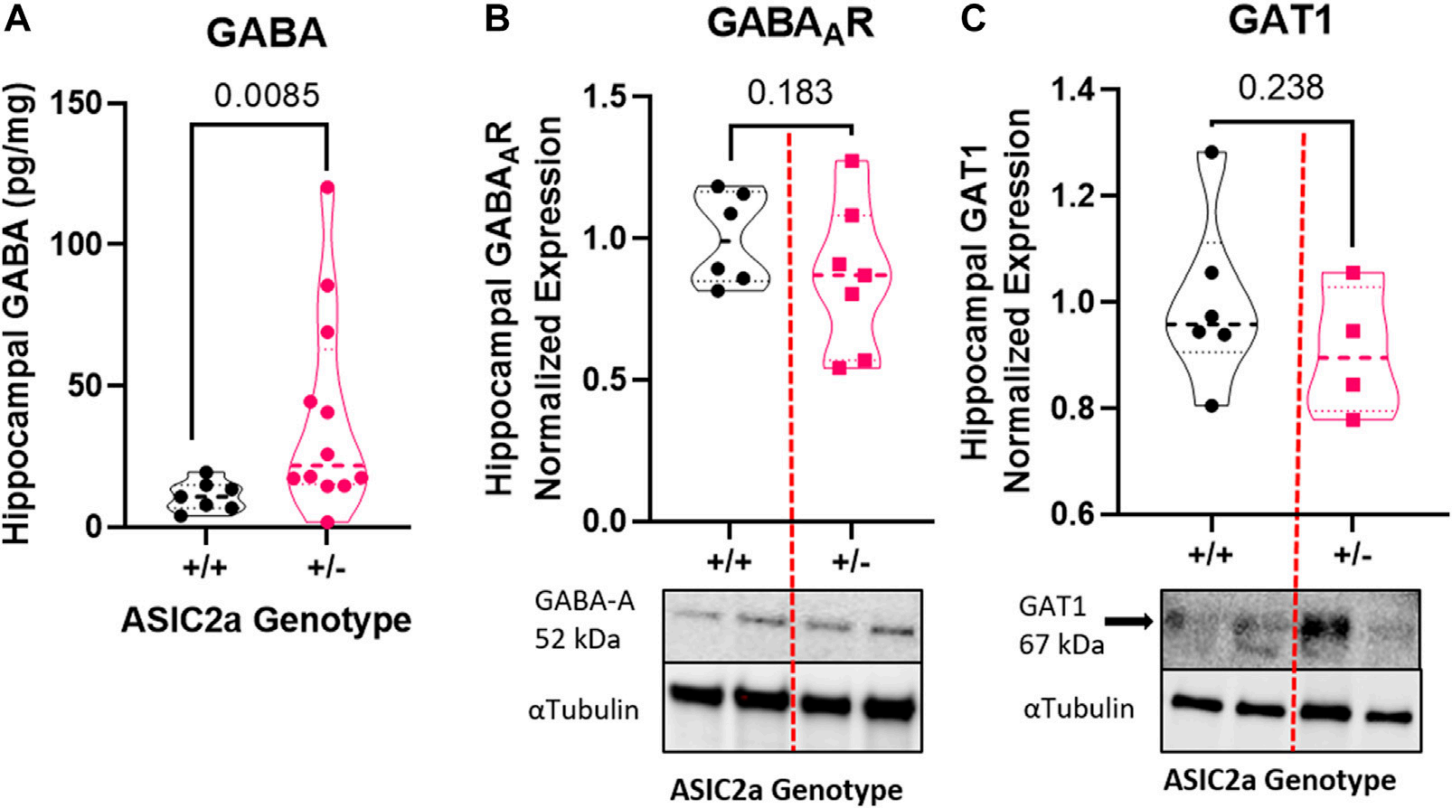
Changes in inhibitory neurotransmitter-related proteins in hippocampal synaptosomes following seizures in pregnant mice with reduced ASIC2a. **(A)** Increased hippocampal synaptosome levels of GABA (*n* = 7 or 14 mice per genotype), and no difference in **(B)** GABA_A_R, and **(C)** GAT1 protein expression following seizures in pregnant ASIC2a heterozygous mice. GABA—γ-Aminobutyric acid, GAT1—GABA transporter 1. N = four to seven mice per genotype. Individual data points are displayed. Thick dashed line is the median and the thin dashed lines are the quartiles Significance was calculated using Mann-Whitney *U* test.

To determine whether seizure exposure is associated with dysregulated GABA response in pregnant ASIC2a +/- mice, we measured the concentration of GABA and the expression of its receptor and transporter in hippocampal synaptosomes. Compared to +/+ mice, there was a significant increase in GABA concentration in +/- mice (11.1 ± 5.3 vs. 39.1 ± 35.5 pg/mg; Figure 3A) in hippocampal synaptosomes. There was no corresponding difference in GABA_A_R (1.0 ± 0.16 in +/+ vs. 0.86 ± 0.26 in +/-; Figure 3B) or GAT1 (1.0 ± 0.16 vs. 0.91 ± 0.12; Figure 3C) expression.

### Acid sensing ion channel 2a HET mice have abnormal inflammatory response to seizures

**FIGURE 4:**
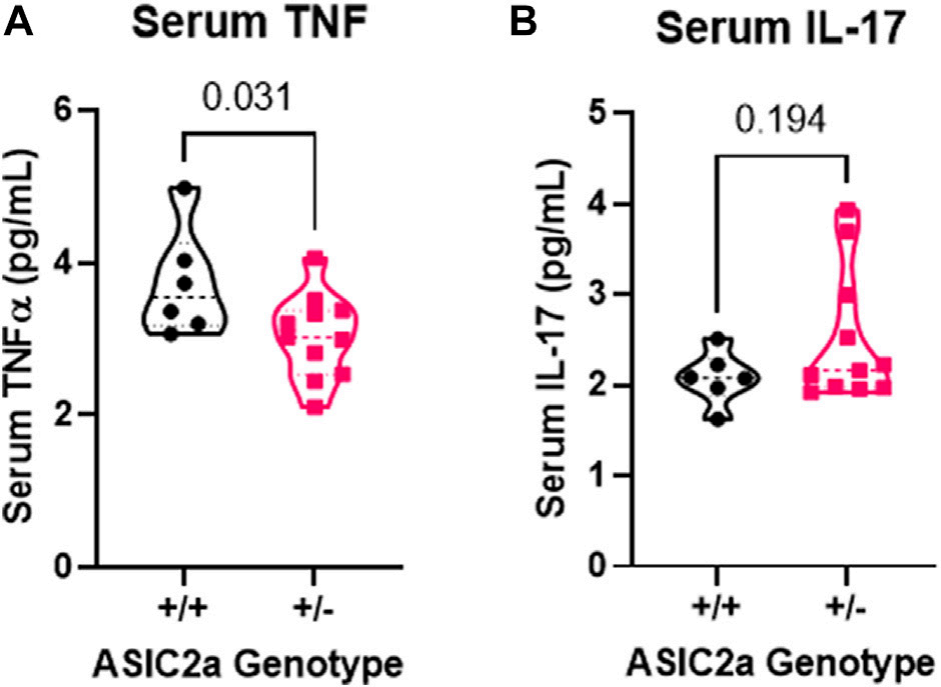
Changes in circulating pro-inflammatory cytokines following seizures. **(A)** Decreased serum TNFα and **(B)** no change in IL-17 levels at GD18.5 of pregnancy following 30 min of seizure exposure. N = 7 or 14 per genotype. Individual data points are displayed. Thick dashed line is the median and the thin dashed lines are the quartiles Significance was calculated using Mann-Whitney *U* test.

Following seizure activity, an inflammatory response is triggered. We therefore measured systemic changes in 2 pro-inflammatory cytokines, TNFα and IL-17 reported to be increased post-seizure. Contrary to our hypothesis that +/- mice will have an exaggerated response, we found a significant reduction in serum TNFα (Figure 4A) and no difference in IL-17 levels (Figure 4B) following acute seizure exposure.

### Pregnant mice with reduced acid sensing ion channel 2a have abnormal hippocampal inflammatory response to seizures

**FIGURE 5:**
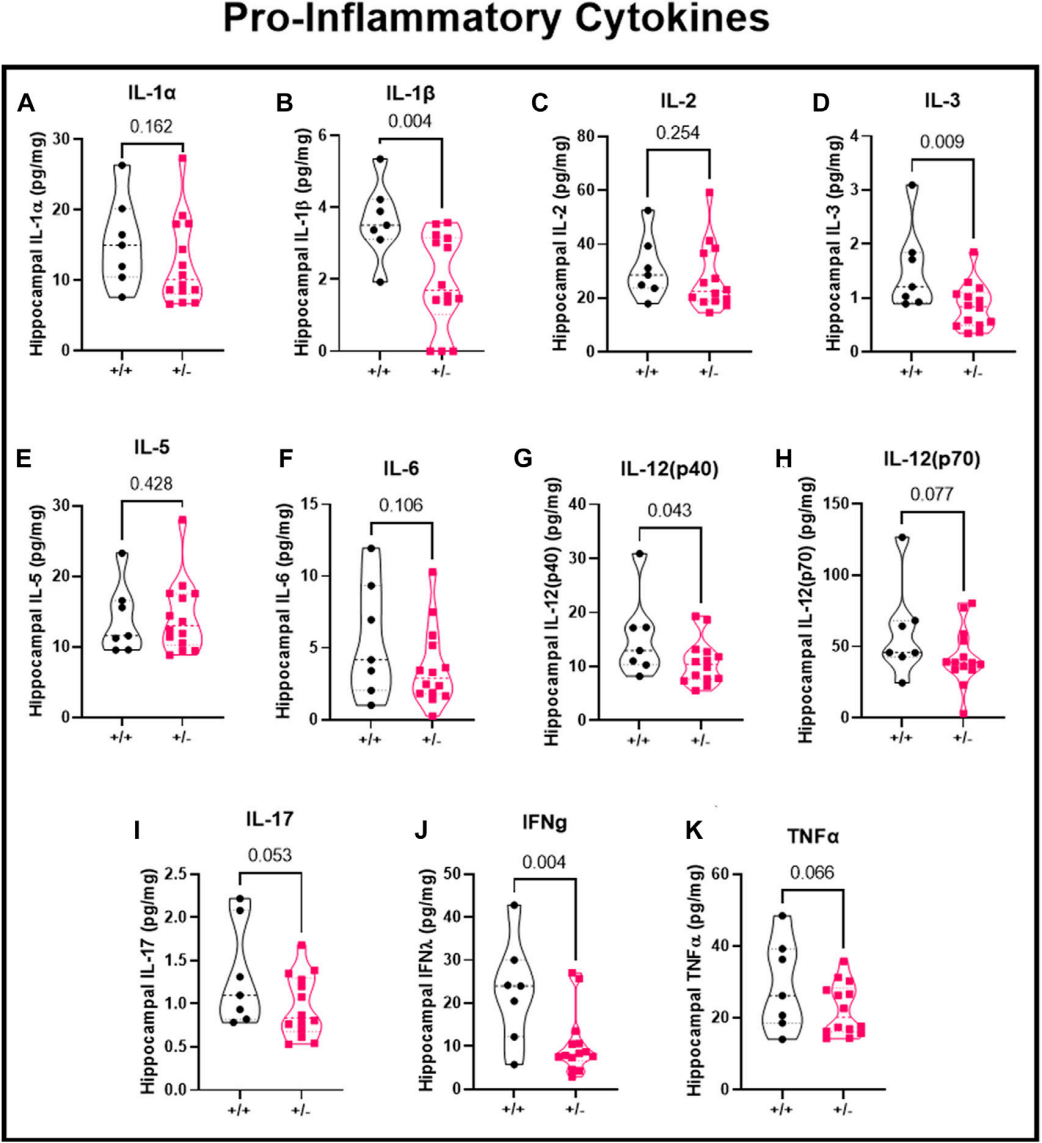
Changes in hippocampal pro-inflammatory cytokines following seizures. Seizure exposure resulted in **(A)** no change in IL-1α, **(B)** decreased IL-1β, **(C)** no change in IL-2, **(D)** decreased IL-3, **(E)** no change in IL-5, **(F)** no change in IL-6, **(G)** no change in IL-12 (p40), or **(H)** IL-12 (p70), or **(I)** IL-17, **(J)** decreased IFNγ, and **(K)** no change in TNFα in hippocampal cytosolic fraction at GD18.5 of pregnancy. N = 7 or 14 per genotype. Individual data points are displayed. Thick dashed line is the median and the thin dashed lines are the quartiles Significance was calculated using Mann-Whitney *U* test.

Using the hippocampal cytosolic fraction and a multiplex assay, we investigated the local pro-inflammatory cytokines in the hippocampus following seizure activity. Inflammatory response were separated based on pro-inflammatory cytokines, anti-inflammatory cytokines, and chemokines. Following seizures, there was no difference in IL-1α (15.4 ± 6.3 vs. 12.6 ± 6.1 pg/mg; Figure 5A), reduced IL-1β (3.6 ± 1.1 vs. 1.9 ± 1.3 pg/mg; Figure 5B), no change in IL-2 (31.1 ± 11.6 vs. 27.3 ± 12.4 pg/mg; Figure 5C), decreased IL-3 (1.5 ± 0.8 vs. 0.9 ± 0.4 pg/mg; Figure 5D), no change in IL-5 (13.9 ± 5.0 vs. 14.4 ± 5.1 pg/mg; Figure 5E), or IL-6 (5.5 ± 4.0 vs. 3.6 ± 2.7 pg/mg; Figure 5F), decreased IL-12 (p40) (15.4 ± 7.6 vs. 10.8 ± 4.2 pg/mg; Figure 5G), IL-12 (p70) (59.7 ± 32.8 vs. 42.6 vs. 20.0 pg/mg; Figure 5H), IL-17 (1.3 ± 0.6 vs. 1.0 ± 0.4 pg/mg; Figure 5I), Interferon gamma (22.8 ± 12.0 vs. 10.5 ± 7.3 pg/mg; Figure 5J), and TNFα (29.1 ± 12.6 vs. 22.3 ± 7.2 pg/mg; Figure 5K) in ASIC2a +/+ vs. +/- pregnant mice. IL-9 levels were below detectable range and not shown in figure.

### Seizure activity leads to reduced hippocampal chemokines in pregnant mice with reduced acid sensing ion channel 2a

**FIGURE 6:**
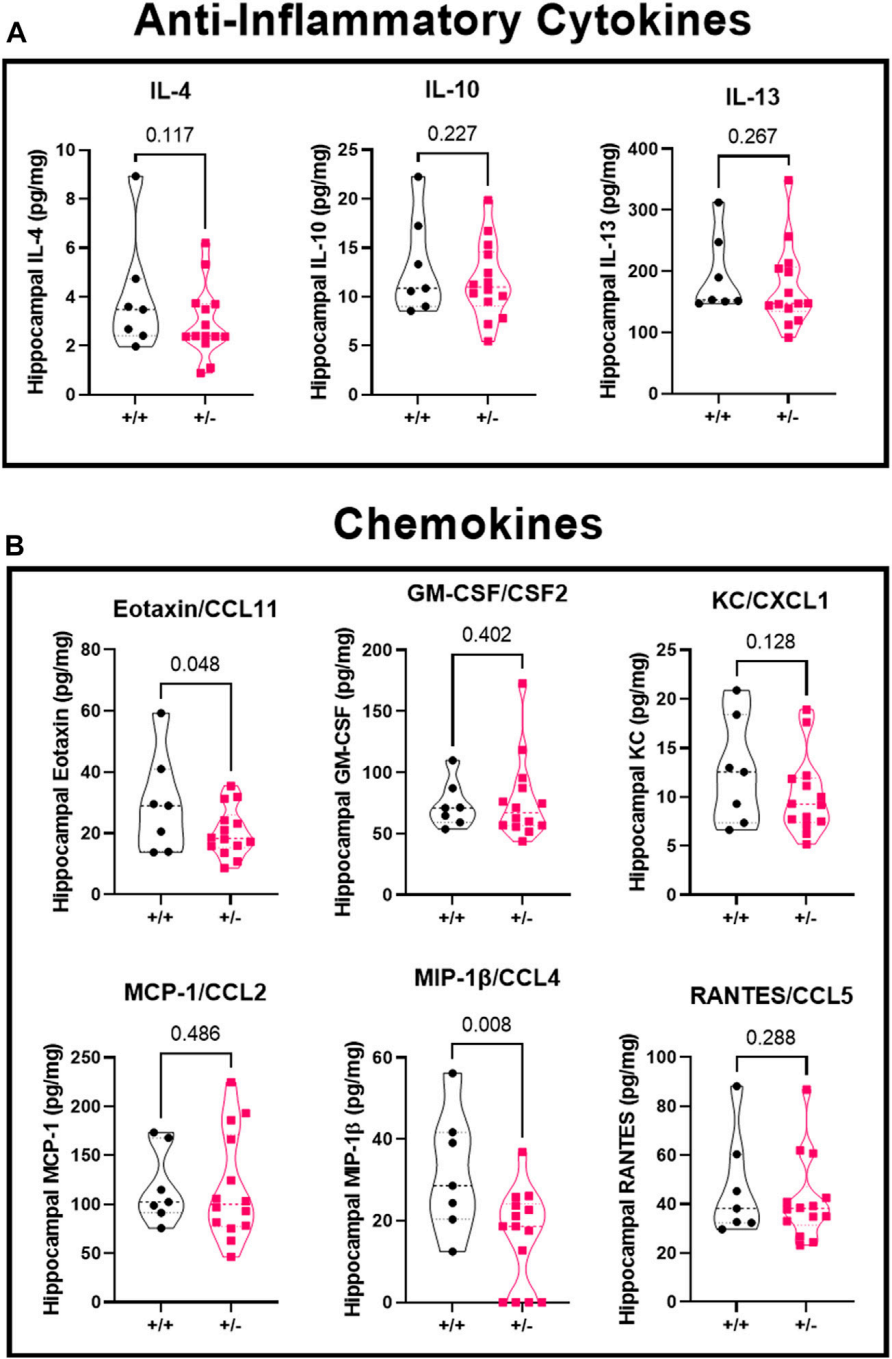
Changes in hippocampal anti-inflammatory cytokines and chemokines following seizures. Seizure exposure led to no differences in anti-inflammatory cytokines **(A)**, IL-4, IL-10, or IL-13. Analysis of the chemokines **(B)** shows a reduction in eotaxin/CCL11, no difference in GM-CSF, KC, or MCP-1, a decrease in MIP-1β, and no change in RANTES levels at GD18.5 of pregnancy after 30 min of seizure exposure. N = 7 or 14 per genotype. Individual data points are displayed. Thick dashed line is the median and the thin dashed lines are the quartiles Significance was calculated using Mann-Whitney *U* test.

There was no significant difference in concentration of anti-inflammatory cytokines (Figure 6A) IL-4 (4.0 ± 2.4 vs. 2.9 ± 1.5 pg/mg), IL-10 (13.1 ± 5.0 vs. 11.6 ± 3.9 pg/mg), or IL-13 (193.2 ± 63.9 vs. 173.8 ± 67.1 pg/mg) in ASIC2a +/+ vs. +/- mice. When investigating chemokine concentration (Figure 6B), we found a significant reduction in Eotaxin/CCL11 (29.6 ± 16.3 vs. 20.4 ± 8.0 pg/mg), no difference in GM-C SF/CSF2 (73.9 ± 19.1 vs. 77.4 ± 33.8 pg/mg), KC/CXCL1 (12.6 ± 5.4 vs. 10.1 ± 4.0 pg/mg), MCP-1/CCL2 (117.8 ± 38.0 vs. 117.0 ± 54.3 pg/mg), a reduction in MIP-1β/CCL4 (31.8 ± 14.8 vs. 16.0 ± 11.8 pg/mg), and no difference in RANTES/CCL5 (46.7 ± 21.1 vs. 41.8 ± 17.2 pg/mg) in ASIC2a +/- mice. MIP-1α and G-CSF levels were below detectable range and are not shown.

### There was no difference in microglia activation following seizures in pregnant mice with reduced acid sensing ion channel 2a

**FIGURE 7:**
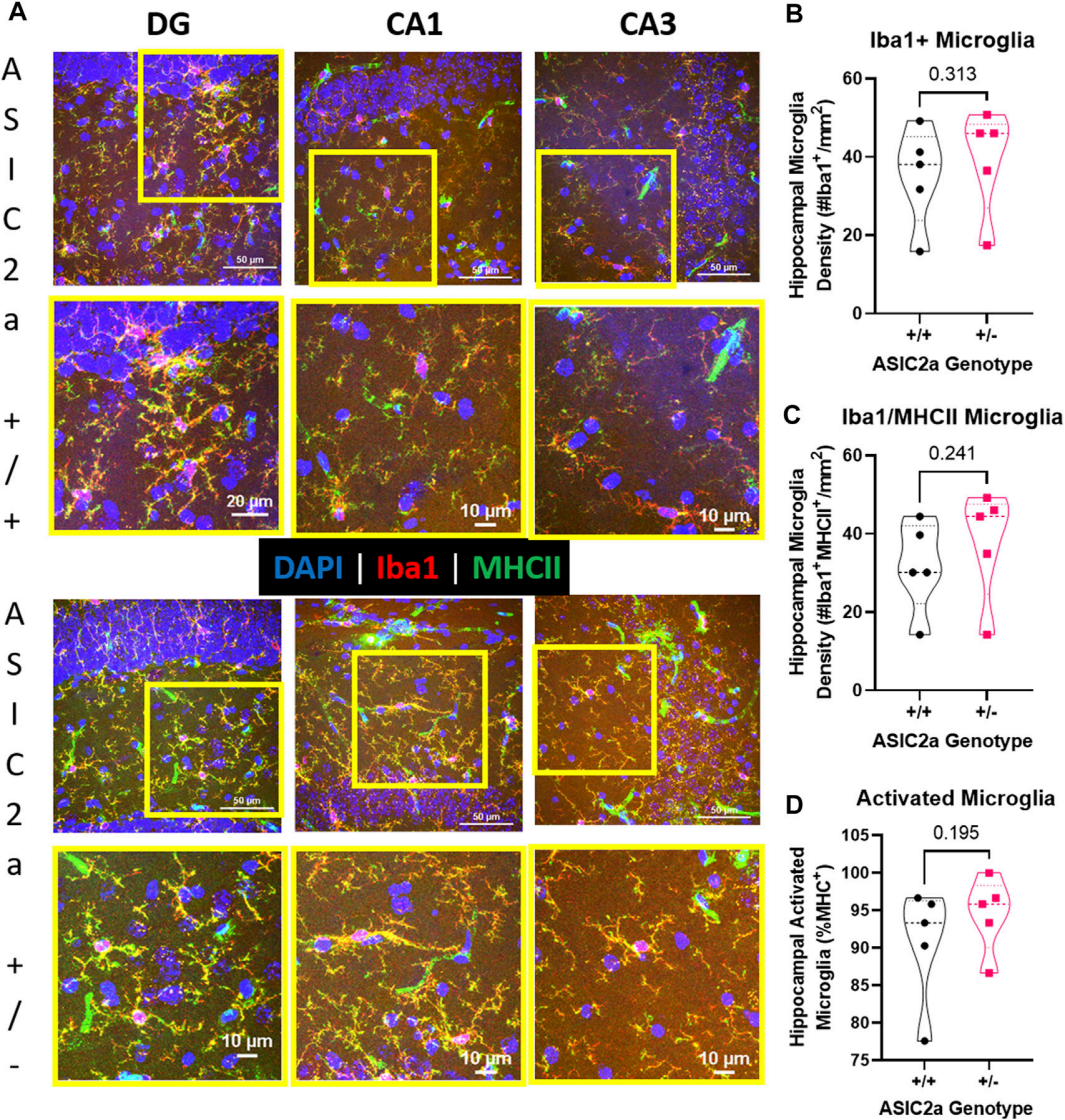
Hippocampal microglia changes following acute seizure exposure. **(A)** Representative images from dentate gyrus (DG), CA1, and CA3 are shown for ASIC2a +/+ and ASIC2a +/- mice. Yellow squares represent the selected field for the higher magnification images (shown below). Majority of the microglia co-expressed major histocompatibility complex II (MHCII). Scale bars represent 50 µm or 10 µm. Microglia density was determined in images from dentate gyrus, CA1, and CA3 and averaged to obtain hippocampal microglia density based on the expression of **(B)** Iba1, **(C)** Iba1/MHCII, and **(D)** percentage of Iba1/MHCII to total Iba1+ microglia. Individual data points are displayed. Thick dashed line is the median and the thin dashed lines are the quartiles Significance was calculated using Mann-Whitney *U* test.

Following seizures, microglia are activated. We used immunofluorescence staining to identify microglia in the hippocampus of ASIC2a +/+ vs. +/- mice. Representative images of microglia are shown in Figure 7A. Microglia were counted in the dentate gyrus, CA1 and CA3 regions of the hippocampus and averaged to determine hippocampal density. There was no difference in the density of Iba1+ microglia (35 ± 13 vs. 39 ± 13; Figure 7B), or microglia co-expressing MHCII (32 ± 12 vs. 38 ± 14; Figure 7C). Lastly, there was no difference in the percent of activated microglia (90.7 ± 7.8 vs. 94.5 ± 5.0%; Figure 7D) in the hippocampus of ASIC2a +/+ vs. +/- mice.

## Discussion

The mechanisms for increased seizure sensitivity in pregnancy and preeclampsia are not fully elucidated. While we have shown that the reduced utero-placental perfusion pressure (RUPP) rat model has increased seizure sensitivity and cerebrospinal fluid inflammatory cytokines at late pregnancy (Warrington, 2015), the underlying molecular mechanisms have not been established. Our initial assessment of the mouse RUPP model revealed a significant decrease in both placental and hippocampal levels of ASIC2a (Jones-Muhammad et al., 2021). Furthermore, when ASIC2a +/- pregnant mice were subjected to seizures, we showed that seizure severity and sensitivity was significantly increased compared to the pregnant +/+ control mice (Jones-Muhammad et al., 2021). Thus, we now further investigated whether ASIC2a +/- mice had a differential molecular signature in relation to neurotransmitter activity at the synapse and inflammatory response (systemic and hippocampal). We show that pregnant ASIC2a +/- mice have no apparent changes in glutamate concentration, receptor or transporter expression in synaptosomes isolated from the hippocampus. Interestingly, GABA concentration was increased in hippocampal synaptosomes, with no change in GABA receptor or transporter expression. We also report that contrary to our hypothesis, pregnant ASIC2a +/- mice subjected to seizures have decreased concentration of serum TNFα and a significant decrease in hippocampal pro-inflammatory cytokines and chemokines. Lastly, we report that there is no difference in hippocampal microglia density or activation state in pregnant ASIC2a +/- mice post-seizure.

### Acid sensing ion channel 2a and seizures

Our current study builds on our findings of increased seizure severity and sensitivity in mice with reduced ASIC2a, a finding that challenged another study reporting worse seizures in male rats with ASIC2a overexpressed in the hippocampus (Wu et al., 2016; Zhang et al., 2017). We surmised that the differences in outcomes could have been driven by the fact that different species and sexes were studied, with our study including an added complexity of pregnancy. Other ASIC isoforms have also been investigated in the context of seizures or epilepsy. Guo et al. (2014) reported that ASIC1 expression was downregulated in cortical lesions of epilepsy patients. Moreover, Liang et al. demonstrated that agonizing ASIC1a receptors reduced seizure latency and seizure score (Liang et al., 2015), and Cao et al. (2016) showed a similar pattern for ASIC3, where increased expression reduced seizure activity. Although the expression or activity of these ASIC isoforms were shown to be inversely associated with seizure activity, the role of ASIC2a in seizure activity is less clear. We have not yet determined whether non-pregnant female mice have the same behavioral response to seizures and whether the pregnancy response in ASIC2a +/- mice is more pronounced than the +/+ control. Elevated ASIC2a expression in hippocampal samples was observed in patients with a history of epilepsy (Zhang et al., 2017). Because ASIC2a was measured in patients with established epilepsy, it is not known if ASIC2a levels gradually increased as the epilepsy disorder progressed or whether ASIC2a levels were already different before the development of the seizure disorder. Thus, the reported increase in ASIC2a in epilepsy patients may be applicable to women who have epilepsy before becoming pregnant rather than *de novo* seizures during gestation or in the postpartum period (Zhang et al., 2017). We have not assessed whether ASIC2a expression increases following seizures similar to what is observed in epilepsy patients. This question will be investigated in future studies.

### Acid sensing ion channel 2a and neurotransmitter activity changes

ASIC1a and ASIC2a localize to the synapse and dendritic spines of neurons (Cao et al., 2016). Here, we show no significant differences in hippocampal glutamate levels at the synapse of ASIC2a +/- compared to +/+ mice following seizures. There was also no change in NMDAR1 or NMDAR2b, or the astrocytic transporters responsible for glutamate reuptake (EAAT1 and EAAT2). We did not assess changes in NMDAR2a expression in this study but acknowledge that we cannot rule out possible changes in NMDAR2a expression. In a previous study, NMDAR2a was co-expressed with NMDAR2b, and both isoforms were significantly increased in kindled rat hippocampus and temporal lobe epilepsy patients (Mathern et al., 1998). Previous work reported a significant increase in glutamate concentration following seizures; however, this significant increase was observed only after chronic exposure to seizure induction, rather than acute exposure like in the current work (Kolosowska et al., 2016; Batten et al., 2017). To our knowledge, our study is the first to report changes in hippocampal synaptosome glutamate-related proteins in pregnant mice following acute seizure exposure.

The relationship of GABA and glutamate activity during seizure has been challenged in recent work, where Losi and colleagues suggest hyperactivity of GABA interneurons precedes increased glutamatergic activity during seizure generation (Losi et al., 2021). Work by MacMullin and colleagues shows that a mouse model of traumatic brain injury displays an increase in the ratio of Glutamate/GABA, which was associated with increased sensitivity to seizures (MacMullin et al., 2020). Because of this recent challenge to the glutamate/GABA relationship in seizure activity, the increase in GABA concentration observed in ASIC2a +/- mice could suggest early activation of GABA interneurons and thus an earlier reaction to seizure exposure compared to ASIC2a +/+ pregnant mice. Additionally, previous work has investigated the association of ASICs with GABA_A_R activity. GABA_A_R activation inhibited ASIC currents in the hippocampus (Chen et al., 2011). Because this study does not specify which isoforms of ASIC may be more associated with GABA_A_R activation, it is not clear how ASIC2a may be affected. Because this study analyzed changes in current amplitude at pH 5.8 and ASIC2a activation is around 4.5 (Kweon and Suh, 2013), it is possible that ASIC2a currents may not be altered by GABA_A_R activation in this particular study. However, given that ASIC2a forms heterodimers with ASIC1a, which has a pH activation threshold around 6.95 (Cristofori-Armstrong and Rash, 2017), GABA_A_R may affect current amplitudes of this dimer. What’s more, ASIC2a is associated with proper trafficking of ASIC1a to the cell membrane (Askwith et al., 2004). This suggests reduced ASIC2a expression may interfere with the ability of ASIC1a to respond to seizure activity. Exploring whether ASIC2a +/- mice have reduced cell trafficking of ASIC1a or reduced currents associated with ASIC1a activation will better explain whether this phenomenon is associated with the mice in the current study having increased seizure sensitivity.

### Reduced acid sensing ion channel 2a and abnormal inflammatory response

The current work shows ASIC2a +/- mice have an abnormal inflammatory response to PTZ-induced seizures, where pro-inflammatory cytokine as well as chemokine concentration is reduced in the hippocampus with no difference in concentration of anti-inflammatory cytokines. Additionally, we did not observe a significant difference in the number of activated microglia present in ASIC2a +/- mice after seizure exposure. Because seizure activity is associated with increased neuroinflammation (De Simoni et al., 2000; Galic et al., 2008; Vezzani et al., 2011), it was surprising to find ASIC2a +/- mice observed reduced inflammatory responses compared to the +/+ control mice.

ASICs are expressed on both neurons and astrocytes (Huang et al., 2010), where ASIC1, ASIC2a, and ASIC3 were found in higher expression in nuclei of astrocytes, rather than the cell membrane. Because each of these isoforms are also found on microglia, it is no surprise that ASICs are involved in microglia-associated inflammatory responses. Work by Wei Yu and colleagues observed the expression of each ASIC isoform on rat microglia, and reported that ASIC1 was the most abundant isoform present (Yu et al., 2015). ASIC2a was found to be the least expressed in rat microglia, although an inflammatory insult significantly increased ASIC2a expression to levels similar to ASIC1, suggesting that both are important for microglia to respond to inflammatory insults. As mentioned previously and observed elsewhere, ASIC2a is important for proper trafficking of ASIC1a to the cell membrane (Askwith et al., 2004). This suggests that although ASIC2a requires a lower pH for activation, reduced or ameliorated expression of ASIC2a will interfere with the ability of ASIC1a to respond to inflammation and thus microglia will not function properly. This partially explains why ASIC2a +/- mice have abnormal responses to inflammation induced by seizures in the current study. Whether pregnancy also contributed to the abnormal inflammatory response is not clear.

Previous work has established how the state of pregnancy changes peripheral immune responses, such as inflammatory responses shifting from pro-inflammatory to largely anti-inflammatory, in order to prevent the fetus from being targeted by the maternal immune system (Trowsdale and Betz, 2006; Robinson and Klein, 2012). Like the peripheral immune system, the neuro-immune system is also affected by pregnancy. Work by Haim et al. (2017) showed that Iba1+ microglia density was significantly reduced in several regions of the maternal rat brain such as the prefrontal cortex and the dorsal hippocampus during pregnancy and the peripartum period. Additionally, cytokines associated with neuroprotection were significantly increased and microglia were observed to have reduced number of processes, although they were not in the activated state, at late pregnancy stages. This suggests that, like the maternal peripheral immune system, the neuro-immune system is in a less-inflamed state. A similar phenomenon was observed in another study where a lipopolysaccharide challenge in pregnant rats resulted in a suppressed neuro-immune response during pregnancy as well as the postpartum period (Sherer et al., 2017). These observations suggest that reduced expression of ASIC2a is associated with lower inflammation in response to seizures, but pregnancy may be additive to the inflammatory response observed in the current study.

## Conclusion

In conclusion, we show that following acute seizures, pregnant ASIC2a +/- mice have increased concentration of GABA at the synapse independent of changes in glutamate concentration or other glutamatergic components as well as changes in GABAergic components. We also report that pregnant ASIC2a +/- mice have a dampened inflammatory response to PTZ-induced seizures. Our findings are only applicable to acute seizure exposure. We do not know whether longer seizure exposure or repeated seizure exposures would lead to similar findings. Additionally, whether complete knockout using ASIC2a null mice (ASIC2a−/−) would lead to similar findings is not known and will need to be investigated. Nevertheless, exploring how pregnancy and reduced ASIC2a increases sensitivity to seizures will advance our understanding of mechanisms involved in pregnancy-induced seizure conditions such as eclampsia.

## Data Availability

The raw data supporting the conclusion of this article will be made available by the authors, without undue reservation.
